# Dysbiosis of human tumor microbiome and aberrant residence of *Actinomyces* in tumor-associated fibroblasts in young-onset colorectal cancer

**DOI:** 10.3389/fimmu.2022.1008975

**Published:** 2022-09-02

**Authors:** Zhuoqing Xu, Zeping Lv, Fangqian Chen, Yuchen Zhang, Zifeng Xu, Jianting Huo, Wangyi Liu, Suyue Yu, Abudumaimaitijiang Tuersun, Jingkun Zhao, Yaping Zong, Xiaonan Shen, Wenqing Feng, Aiguo Lu

**Affiliations:** ^1^Department of General Surgery, Ruijin Hospital, Shanghai Jiaotong University School of Medicine, Shanghai, China; ^2^Shanghai Minimally Invasive Surgery Center, Ruijin Hospital, Shanghai Jiaotong University School of Medicine, Shanghai, China; ^3^Shanghai Institute of Digestive Surgery, Ruijin Hospital, Shanghai Jiaotong University School of Medicine, Shanghai, China; ^4^Department of Gastroenterology, Ruijin Hospital, Shanghai Jiao Tong University School of Medicine, Shanghai, China

**Keywords:** young-onset colorectal cancer, microbiome, *Actinomyces*, TLR2, cancer-associated fibroblasts (CAFs)

## Abstract

Colorectal cancer (CRC) is the third most common form of cancer, and the incidence of sporadic young-onset colorectal cancer (yCRC) has been increasing. Microbiota residing in the tumor microenvironment are emerging tumor components. The colonic microbiome differs between patients with CRC and healthy controls; however, few studies have investigated the role of the tumor microbiota in disease diagnosis and tumorigenesis of yCRC. We performed 16S rRNA sequencing analysis to identify the microbiome in CRC and found that tumor microbial diversity decreased in yCRC. Proteobacteria and Firmicutes were the most abundant phyla in all CRC samples, and *Actinomyces* and *Schaalia cardiffensis* were the key microbiota in the yCRC group. Correlation analysis revealed that *Actinomyces* co-occurred with various pro-tumor microbial taxa, including Bacteroidia, Gammaproteobacteria, and *Pseudomonas*. An independent cohort was used to validate the results. The *Actinomyces* in CRC was co-localized with cancer-associated fibroblasts and activated the TLR2/NF-κB pathway and reduces CD8+ T lymphocyte infiltration in CRC microenvironment. This study suggests that tumoral microbiota plays an important role in promoting tumorigenesis and therefore has potential as a promising non-invasive tool and intervention target for anti-tumor therapy.

## Introduction

Colorectal cancer (CRC) is one of the most common malignant tumors of the digestive system, and its morbidity and mortality rank third worldwide (1). Traditionally, CRC is considered a disease of elderly individuals, but in recent years, the incidence of sporadic CRC of patients under 50 years of age has been steadily increasing worldwide. Patients with young-onset colorectal cancer (yCRC) often exhibit more advanced disease and adverse pathological features than patients with old-onset CRC (oCRC), which considerably affect the former’s survival outcomes and quality of life (2). There are insufficient clinical diagnostic and therapeutic protocols for yCRC, and the characteristics and mechanisms of tumor progression in yCRC remain unclear.

Tumorigenesis, tumor development, and metastasis are complex processes involving tumor cells and the tumor microenvironment (TME). The microbiota of the TME is an emerging tumor component (3). Increasing evidence suggests that the gut microbiota is involved in the prevention or promotion of different diseases and has emerged as a key environmental factor implicated in the development of CRC (4, 5). The colonic microbiome in patients with CRC and healthy controls differs (6), but few studies have investigated the role of the tumor microbiota in yCRC disease diagnosis and tumor progression. Studies have reported that the gut microbiota of older adults differs from that of younger adults. Owing to lifestyle changes among different generations, the prevalence of major risk factors (e.g., unhealthy diets, obesity, and sedentary lifestyles) in young adults is increasing. These changes may contribute to alterations in the gut microbiota, which interact with the underlying genetic background and lead to diseases such as CRC (7). Simultaneously, specific gut bacteria can invade CRC tissues and alter the TME (8). Therefore, there may be a characteristic spectrum of pathogenic microbiota with diagnostic value for yCRC.

Ma et al. performed 16S rRNA gene sequencing for 1038 samples from China and analyzed the fecal microbial composition, functional changes in the microbial community, and microbial markers in yCRC, oCRC, and age-matched healthy controls (9). Their research demonstrated the powerful classification potential of gut microbiota biomarkers for the accurate detection and differentiation of individuals with yCRC. However, owing to differences in the invasive and colonizing abilities of bacteria, the gut microbiota cannot fully reflect the microbial composition in the TME, and few studies have reported the compositional and functional changes in the tumor microbial community in yCRC. Therefore, in this study, we characterized the tumor microbiota of yCRC and oCRC to identify microbial markers for yCRC diagnosis and explored their potential roles in the tumor immune microenvironment and tumorigenesis. We hypothesized that yCRC and oCRC may have distinct tumor microbial bases and play an important role in promoting tumorigenesis and tumor progression, potentially serving as promising non-invasive tools and intervention targets for anti-tumor therapy.

## Methods

### Patients and specimens

Specimens from patients with CRC were collected after obtaining informed consent from the Biomedical Ethics Committee of Ruijin Hospital. Between 2020 and 2022, we collected tumor tissues from 39 patients at the Shanghai Minimally Invasive Surgery Center of Ruijin Hospital for 16S rRNA sequencing analysis (discovery cohort) (**Tables S1**, **S7**). A tissue microarray consisting of 78 pairs of CRC samples [validation set; described in our previous study (10)] was used for Immunohistochemistry (IHC) staining (**Table S2**). All patients were pathologically diagnosed with CRC and underwent laparoscopic surgery at our center. Sections from each patient were TNM staged according to the 2015 National Comprehensive Cancer Network guidelines. All patients were fully informed of the study and signed an informed consent form.

### 16S rDNA sequencing

Snap-frozen tumor tissue samples from patients with CRC were sent to TinyGene Bio-Tech (Shanghai) Co., Ltd. and subjected to 16S rDNA sequencing using the Illumina MiSeq platform. For details regarding the materials and methods, please refer to the Supplemental Information. The original raw data of 16S rDNA sequencing was uploaded to NCBI SRA database and the BioProject accession number was PRJNA865279.

### Fluorescence *in situ* hybridization

We prepared 4-µm thick tissue sections from paraffin-embedded specimens and dewaxed them. The specimens were then treated with proteinase K solution in a dry oven at 37°C for 30 min. Subsequently, a DNA probe targeting *Actinomyces* was added at 400 nM and incubated overnight in a humid environment at 37–45°C. The slides were mounted and observed under a microscope. Details of the probe sequence are provided in **Table S3**. For the 16S rDNA sequencing, samples with *Actinomyces* detectable were defined as high *Actinomyces* abundance, and samples below detectable abundance were defined as low *Actinomyces* abundance. For the FISH analysis, the *Actinomyces* positive staining area at 10% as the cut-off value between high *Actinomyces* abundance and low *Actinomyces* abundance.

### IHC analysis

A tissue microarray consisting of 78 pairs of CRC samples, which was described in our previous study, was used for IHC staining. IHC staining was performed according to the manufacturer’s protocol (Immunostain SP Kit, DakoCytomation, USA). In brief, the tissues were fixed in 4% neutral buffered formalin, paraffin-embedded, and sectioned. The slices were IHC stained, and TLR2, TLR4, CD45, CD8, NF-κB, and α-SMA antibodies were used for IHC analysis. IHC staining was scored semi-quantitatively by two independent pathologists. Details on the antibodies are provided in **Table S4**.

### Bioinformatics analysis

The correlation between the mRNA expression of TLR2, TLR4, and NF-κB was analyzed using GEPIA (http://gepia.cancer-pku.cn/). The infiltration of immune cells into the tumor was analyzed using TIMER (http://timer.cistrome.org/).

### Statistical analyses

Quantitative data were compared using the Student’s t test. The associations between clinical characteristics were assessed using Pearson’s chi-square test or Fisher’s exact test. Data are shown as the mean ± SD. Statistical significance was set at P < 0.05. All data were analyzed using SPSS version 20.0 (IBM Corp., Armonk, NY, USA), Graph Pad Prism 8.0 (Graph Pad software, lnc., San Diego, CA, USA), R version 3.6.3 (R Foundation for Statistical Computing, Vienna, Austria), and Microsoft Excel (Microsoft Corporation, Seattle, WA, USA).

## Results

### Participant characteristics

We included 39 individuals undergoing radical colon cancer surgery in the current analysis: 20 patients with yCRC and 19 in patients with CRC (**Table S1**). Patients with CRC with a family history of cancer were excluded. The patients with yCRC and oCRC were similar in sex, Body Mass Index (BMI), and TNM stage. Forty-five percent of yCRC cases (n = 9) and 53% of oCRC cases (n = 10) were considered advanced. A total of 4,776,993 paired-end reads were generated, with an average (s.d.) of 122,487 reads per sample. After quality control, we obtained 3,700,525 high-quality reads free of adaptor and human DNA contaminants, with an average (s.d.) of 94,885 reads per sample (Additional file 1). The obtained valid data were then deduplicated to obtain the deduplication sequence amplicon sequence variants (ASVs) or operational taxonomic units (OTUs). Finally, normalization was performed using the diversity core-metrics-phylogenetic command in QIIME2 software. The normalized sample sequencing depth was 50,659 and the number of ASVs was 21,659 (Additional file 1). The distribution and overlap of the differential ASV/OTUs between the yCRC and oCRC groups are shown as a Venn diagram (**Figure S1A**). Of a total of 20,114 ASV/OTUs, 2893 were shared between the two groups, 7061 ASV/OTUs were unique to yCRC, and 10,160 ASV/OTUs were unique to oCRC. We performed rarefaction analysis to determine the rationality of the sequencing data. The rarefaction curve for all samples tended to be flat, suggesting that the sequencing depth was sufficient to capture most of the gene diversity (**Figure S1B**).

### Tumor microbiota diversity of yCRC and oCRC

**Figure 1:**
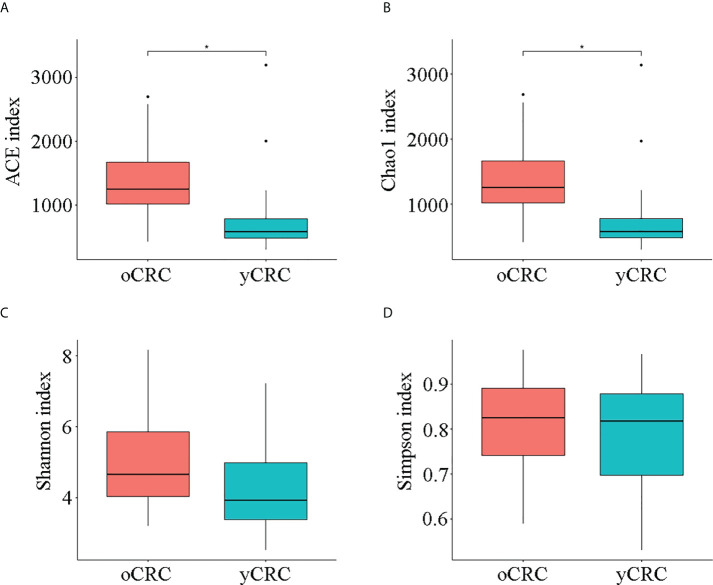
Diversity of tumor microbiota in patients with old-onset colorectal cancer (oCRC) and patients with young-onset colorectal cancer (yCRC). **(A–D)** Alpha diversity of the two groups was measured in terms of the ACE, Chao1, Shannon, and Simpson indices. Data represent the mean ± SD. *P < 0.05.

**Figure 2:**
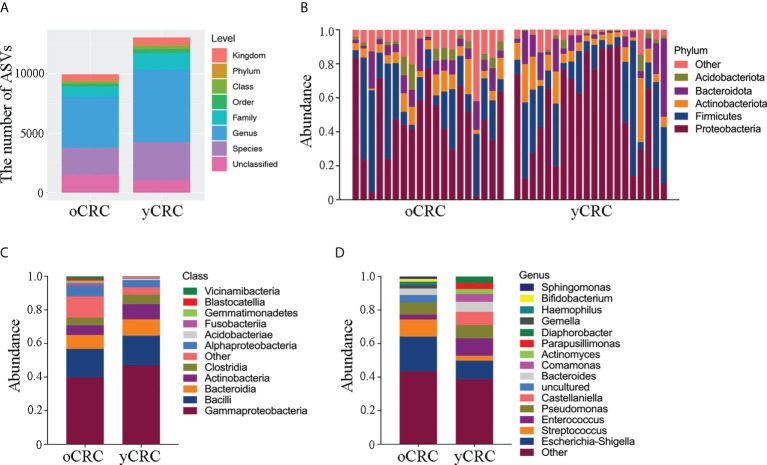
Abundance and diversity of tumor microbiota in patients with oCRC and patients with yCRC. **(A)** Statistical chart of microbial diversity results of each species level by observed count of amplicon sequence variants (ASVs). **(B)** Bar plots displaying taxonomic composition at the major phyla of each sample. **(C, D)** Relative abundance of the tumor microbiota in the two groups at class level and genus level.

We first investigated the microbial diversity of the participants by calculating the alpha diversity of the samples, including microbiota richness and diversity. Microbiota richness was measured according to the Chao1 and abundance-based coverage estimator (ACE) indices to estimate the number of microbiota (**Figures 1A, B**), and microbiota diversity was measured using the Shannon, Simpson, and Pielou_e indices to estimate the evenness of the microbiota (**Figures 1C, D**; **S1C, S1D**). yCRC cases tended to have lower community richness than oCRC cases (ACE index: p = 0.003; Chao1 index: p = 0.002), and the community diversity was similar in the two groups (Shannon index, p = 0.063; Simpson index, p = 0.673; Pielou _e index, p = 0.354) (**Table S5**). The 21,659 ASVs obtained in the first part were aligned with the Silva database (Release138, http://www.arb-silva.de) for species annotation. Sequences were then aligned for alpha diversity to assess differences in bacterial diversity among groups. The results showed that tumor microbial alpha diversity was significantly lower in the yCRC group than in the oCRC group, indicating microflora dysbiosis in yCRC (**Figure 2A**).

To further explore the features of the tumor microbial community in patients with CRC, we compared the relative abundance of the tumor microbiota at the phylum level between yCRCs and oCRCs (**Figures 2B**; **S2A**). Similar phyla were detected in patients with yCRC and patients with oCRC. In all CRC samples, we found that the dominant phyla consisted of Proteobacteria, Firmicutes, Actinobacteria, Bacteroidetes, and Acidobacteria. In contrast, at the class and genus levels, four microbiotas were enriched in patients with yCRC compared to the oCRC group: Gammaproteobacteria, Actinobacteria, *Enterococcus*, and *Castellaniella*, and three microbiotas were reduced in patients with yCRC: Alphaproteobacteria, *Escherichia*-*Shigella*, and *Streptococcus* (**Figures 2C, D**). At the order, family, and species levels, seven microbiotas were enriched in patients with yCRC compared to the oCRC group: Burkholderiales, Alcaligenaceae, Comamonadaceae, Enterococcaceae, Enterococcus_cecorum, Castellaniella_defragrans, and Bacteroides_vulgatus, and six microbiotas were reduced in patients with yCRC: Enterobacterales, Enterobacteriaceae, Streptococcaceae, uncultured_bacterium, Campylobacter_showae, and Treponema_medium (**Figures S2B–D**).

### Identification of specific microbial taxa changes in yCRC

**Figure 3:**
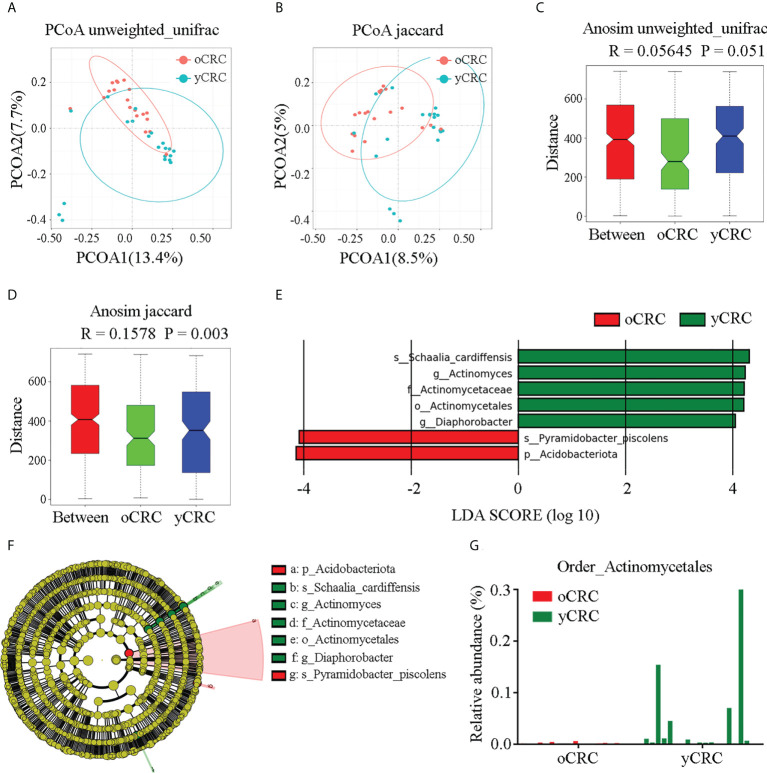
Identification of specific microbial taxa changes in yCRC. **(A, C)** Beta diversity calculated by principal coordinates analysis (PCoA) of Unweighted UniFrac methods and analysis of similarities (Anosim). **(B, D)** Beta diversity calculated by PCoA of Jaccard methods and Anosim, indicating a different distribution of microbial community between oCRC and yCRC. **(E)** The histogram represents linear discriminant analysis (LDA) scores of bacteria with significant differential abundance between two groups. LDA score > 2.0, p < 0.05. **(F)** Taxonomic cladogram represents linear discriminant analysis effect size (LEfSe) analysis for tumor microbiota in two groups. Each node represents a specific taxonomic type. Yellow nodes denote the taxonomic features not significantly differentiated between two groups. Red nodes denote taxonomic types with more abundance in oCRC group than in the yCRC group; green nodes denote taxonomic types more abundant in the yCRC group than in the oCRC group. **(G)** Relative abundance of Actinomycetales of each sample at order level.

To compare the similarity in microbiome diversity between yCRC and oCRC, we performed beta diversity analysis. The distances between samples were calculated using weighted (weighted UniFrac) and unweighted (Jaccard and Unweighted UniFrac) methods, and principal coordinate analysis (PCoA) was performed (**Figures 3A, B**; **S3A–C**). Analysis of similarities (Anosim) was used to verify the significance of the differences in beta diversity among groups of samples. The results showed that the between-sample variability (beta diversity) of the tumor microbial community structure tended to be lower in oCRC than in yCRC (yCRCs *vs*. oCRCs: unweighted UniFrac p = 0.051, Jaccard p = 0.003), suggesting that patients with yCRC have unique diversity and microbial distance (**Figures 3C, D**).

To identify differentially abundant taxa in oCRC and yCRC, we used linear discriminant analysis (LDA) and linear discriminant analysis effect size (LEfSe) algorithms to examine the tumor microbiota compositions of the two groups based on the results of rRNA sequencing analysis, and a logarithmic LDA score cut-off>4.0 was used. The abundance of *Schaalia_cardiffensis*, *Diaphorobacter*, *Actinomyces*, Actinomycetaceae, and Actinomycetales increased in the yCRC group, and the abundance of phylum_Acidobacteriota and species_Pyramidobacter_piscolens increased in the oCRC group (**Figure 3E**). Because Actinomycetales, Actinomycetaceae, *Actinomyces*, and *Schaalia cardiffensis* contained the key phylotypes in the yCRC group, we further identified *Actinomyces* as the key microbiota in the yControl group (**Figure 3F**). To further verify the results of the LDA and LEfSe algorithms, we compared the relative abundance of specific microbiota between the patients with yCRC and patients with oCRC. At the order level, patients with yCRC showed higher prevalence rates of Actinomycetales than those with oCRC (**Figure 3G**). The relative abundances of the specific microbiota in the two groups at the phylum, class, and species levels are shown in **Figures S3D–F**. The statistical distribution and relative abundance of specific microbiota supported the prevalence of *Actinomyces* in most yCRC samples. Collectively, these results show a significant alteration in tumor microbial diversity in yCRC, with a significantly higher abundance of *Actinomyces*.

### Association between *Actinomyces* and microflora dysbiosis in yCRC

**Figure 4:**
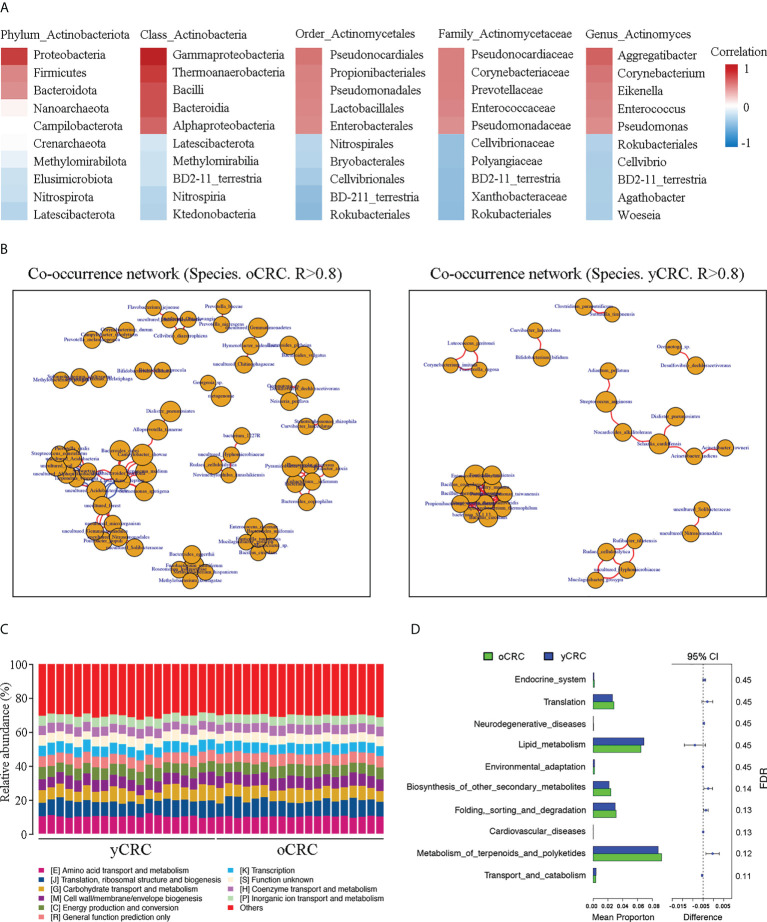
Correlation of *Actinomyces* with differentially abundant genera and functional prediction of the tumor microbiome in CRC. **(A)** Correlation heatmap. Red indicates positive correlations; blue indicates negative correlations. **(B)** Co-occurrence network of oCRC and yCRC. **(C)** PICRUSt analysis identified 10 core predicted categories present in all CRC samples. **(D)** Gene functions in tumor microbiota in patients with oCRC and patients with oCRC.

We investigated whether microflora dysbiosis in yCRC is correlated with the abundance of *Actinomyces*. Correlation analysis revealed that Actinobacteria were positively correlated with Proteobacteria, Firmicutes, and Bacteroidetes at the phylum level (r > 0.5, P < 0.05). Gammaproteobacteria, Bacilli, and Bacteroidia were strongly positively correlated with Actinobacteria at the class level (r > 0.6, P < 0.05). Pseudomonadales and Lactobacillales were positively correlated with Actinomycetales at the order level (r > 0.5, P < 0.05). Enterococcaceae and Pseudomonadaceae were positively but weakly correlated with Actinomycetaceae at the family level (r > 0.4, P < 0.05). Enterococcus and Pseudomonas were positively but weakly correlated with *Actinomyces* at the genus level (r > 0.4, P < 0.05) (**Figure 4A**). To explore the ecological significance of related microbiota and infer their interaction patterns in different habitats (co-occurrence or co-exclusion), we constructed an association network of dominant microbial taxa according to the 16S species composition profile. The results showed that the OTUs that formed correlated networks of yCRC were significantly fewer than those of oCRC, confirming microflora dysbiosis in yCRC. *S. cardiffensis* co-occurred with various tumor microbiota, and OTUs formed a positive network (**Figure 4B**).

### Functional prediction and analysis of the tumor microbiome in CRC

To investigate the differences in the function of tumor microbial communities in the yCRC and oCRC groups, we used the Phylogenetic Investigation of Communities by Reconstruction of Unobserved States (PICRUSt) approach to map 16S sequences to the genes and pathways that these tumor microbiotas may contain. PICRUSt analysis revealed the functional content of different marker gene sequences and identified 10 core predicted categories present in all CRC samples (**Figure 4C**). Subsequently, we compared the enrichment of differential pathways between the yCRC and oCRC groups; the results showed that yCRC samples exhibited enrichment in predicted functional categories related to metabolic processes, such as lipid transport and metabolism (**Figures 4D**; **S4A**). Our results suggest that patients with yCRC have bacterial metabolic profiles unique from those of patients with oCRC.

### *Actinomyces* resides in cancer-associated fibroblasts and affects the TME

**Figure 5:**
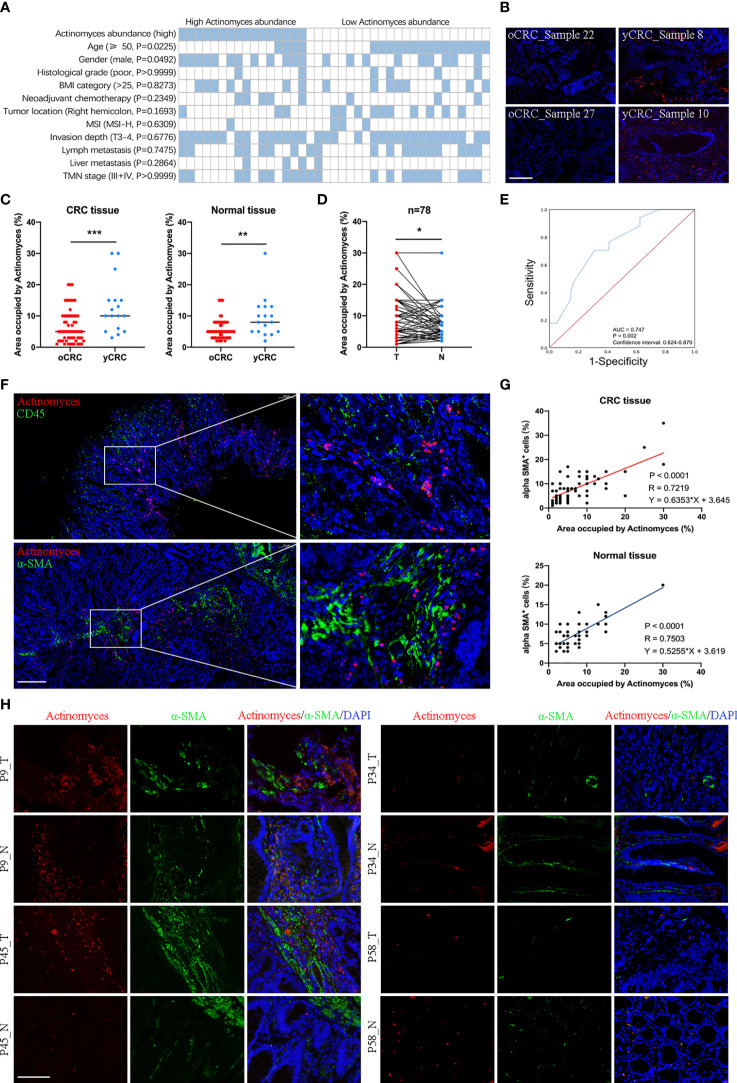
Localization of *Actinomyces* in CRC. **(A)** Heatmap shows clinical characteristics of 39 patients for 16S rRNA sequencing analysis. Statistical significance was analyzed by the χ2 test. P values are as indicated. **(B)** *Actinomyces* staining in human CRC tissues. Fluorescence *in situ* hybridization (FISH) analysis revealed the presence of punctate bacteria (red) in yCRC tissues. Scale, 100 μm. **(C)** The area occupied by *Actinomyces* was evaluated by FISH. Abundance of *Actinomyces* in yCRC was higher than that in oCRC (mean ± SD, Student’s t-test, **P < 0.01, ***, p < 0.001). **(D)** Abundance of *Actinomyces* in CRC tissues was slightly higher than that in normal tissues (mean ± SD, Student’s t-test, *P < 0.05). **(E)** Receiver operating characteristic curves (ROC) of oCRC *vs*. yCRC in validation set (n = 78). Area under the curve (AUC) values for prediction of oCRC and yCRC using *Actinomyces* markers. **(F)** FISH analysis revealed the localization of *Actinomyces* in CRC. Most of the positively stained bacteria were enriched in cancer-associated fibroblasts (α-SMA+), and a few co-localized with immune cells (CD45+). Scale, 200 μm. **(G)** The proportion of α-SMA+ cells was positively correlated with abundance of *Actinomyces* in CRC tissues and normal tissues. **(H)** FISH and IHC staining in human CRC tissues and normal tissues. Red, *Actinomyces* probe. Green, α-SMA. Scale, 100 μm.

To explore the clinicopathological significance of *Actinomyces* in CRC, we performed a more detailed analysis of the clinical data of patients with CRC (**Figure 5A**). Patients with CRC were divided into high and low categories based on the median relative abundance of *Actinomyces*. The analysis showed that a high expression of *Actinomyces* was significantly correlated with age (P = 0.0225) and sex (P = 0.0492). To further validate our results, a tissue microarray consisting of 78 pairs of CRC samples, which was described in our previous study, served as an independent external validation phase. A specific probe of *Actinomyces* was synthesized based on the specific OTU sequence of *Actinomyces* obtained by 16S rRNA sequencing and was used in FISH to target *Actinomyces* regions in CRC tissues. We first validated the specificity of the probes in CRC tissues with high and low *Actinomyces* abundances and found that the percentage of *Actinomyces* positive staining area in the high *Actinomyces* abundance group was higher than 10% (**Figure 5B**). Subsequently, we evaluated whether *Actinomyces* was significantly enriched in CRC and whether *Actinomyces* could be used to discriminate between yCRC and oCRC. The results showed that the abundance of *Actinomyces* in yCRC was higher than that in oCRC (**Figure 5C**), and the abundance of *Actinomyces* in CRC tissues was slightly higher than that in normal tissues (**Figure 5D**). Receiver operating curve (ROC) analysis showed that *Actinomyces* performed well in identifying yCRCs, with an area under the curve (AUC) of 0.747 (**Figure 5E**). In addition, we detected immune cells in the same CRC samples using the CD45 antibody and CAFs. The results showed that most of the positive *Actinomyces* DNA was enriched in CAFs, and a few were co-localized with immune cells (**Figures 5F, H**). Correlation analysis revealed that the proportion of α-SMA+ cells was positively correlated with the abundance of *Actinomyces* in CRC and normal tissues (**Figure 5G**). These results strongly suggest that *Actinomyces* in CRC resides in CAFs and thus affects the TME.

### *Actinomyces* activates the TLR2/NF-κB pathway and reduces CD8+ T lymphocyte infiltration in CRC microenvironment

**Figure 6:**
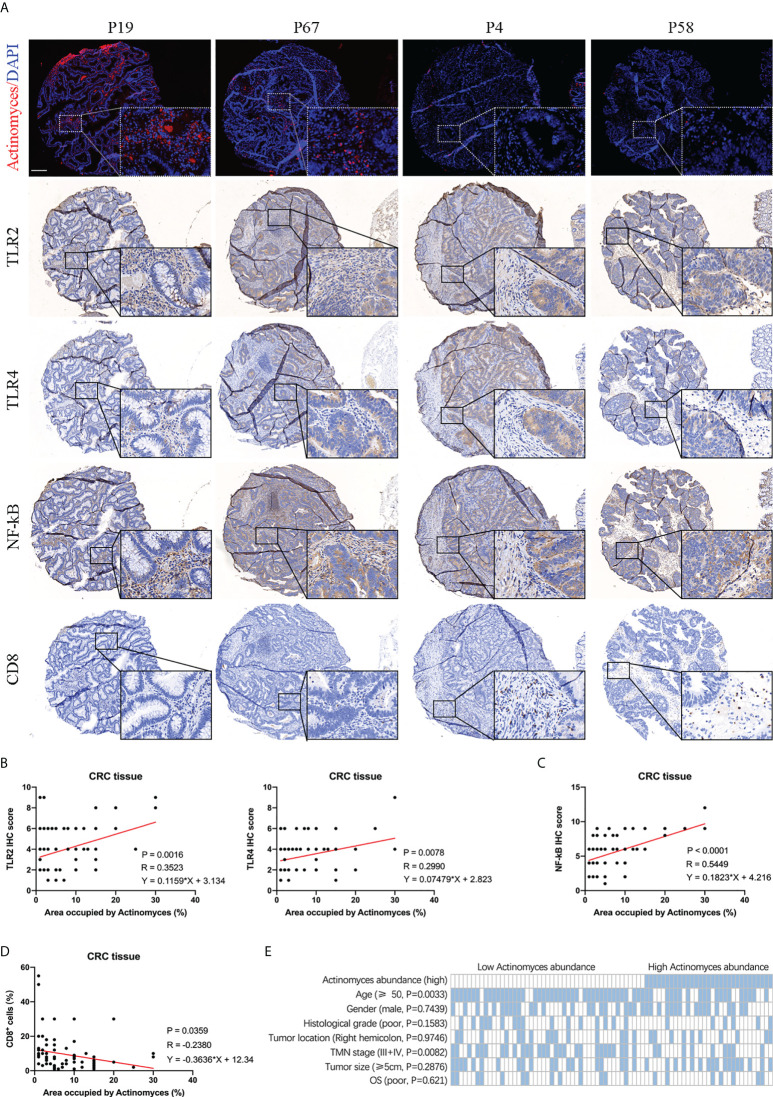
Abundance of *Actinomyces* was associated with TLR2/4 activation and immune cell infiltration in CRC. **(A)** FISH and IHC staining of TLR2, TLR4, NF-κB, and CD8 in human CRC tissues. Scale, 200 μm. **(B)** Expression of TLR2 and TLR4 were positively correlated with abundance of *Actinomyces* in CRC tissues. **(C)** Expression of NF-κB was positively correlated with abundance of *Actinomyces* in CRC tissues. **(D)** Proportion of CD8+ cells was negatively correlated with abundance of *Actinomyces* in CRC tissues. **(E)** Heatmap shows the clinical characteristics of 78 patients for IHC analysis. Statistical significance was analyzed by the χ2 test. P values are as indicated.

Tumor microbiota has been revealed to play an active role in cancer development by recognizing toll like receptors (TLR) and regulating the expression of immune response genes (11). We further investigated whether infection with the gram-positive bacteria *Actinomyces* affects the inflammatory phenotype and anti-tumor immune response of CRC. TLR2 and TLR4 are responsible for the recognition of gram-positive and gram-negative bacteria, respectively (12), and both TLR2 and TLR4 can activate NF-κB signaling and mediate the inhibition of CD8+ T lymphocytes (13). In addition, GEPIA software showed that the expression of TLR2, TLR4, and NF-κB was highly correlated (**Figure S5A**). Subsequently, we detected the expression of TLR2, TLR4, CD8, and NF-κB in CRC tissues by IHC staining (**Figure 6A**). The results showed that expression levels of TLR2, TLR4, and NF-κB were higher in CRCs with high *Actinomyces* abundance than in CRCs with low *Actinomyces* abundance, indicating that *Actinomyces* induced activation of the TLR2/NF-κB pathway (**Figures 6B, C**). Due to the coexistence of *Actinomyces* with Gram-negative bacteria (e.g. Bacteroidia, Gammaproteobacteria, and *Pseudomonas*), TLR4/NF-κB activation also existed in tissues with high abundance of *Actinomyces*. Subsequently, TIMER software was used to analyze the infiltration of immune cells in CRC. The expression of TLR2, TLR4 and NFKB1 were highly positively correlated with the infiltration of neutrophils, macrophages, dendritic cells, and CAFs, while TLR2 was negatively correlated with the infiltration of CD8+ T cells (**Figure S5B**). Notably, the number of CD8+ T lymphocytes was significantly reduced in CRC with a high abundance of *Actinomyces*, suggesting that *Actinomyces* and the ecological environment inhibited the infiltration of CD8+ T lymphocytes into CRC (**Figure 6D**). Finally, we analyzed the clinical characteristics of the discovery cohort of patients with CRC (n = 78). The results show that high expression of *Actinomyces* was significantly correlated with age (P = 0.0033) and TMN stage (P = 0.0082) (**Figure 6E**). In contrast, the abundance of *Actinomyces* was not significantly correlated with the survival time of patients with CRC (**Figure S5C**), suggesting that *Actinomyces* mainly affects tumorigenesis in CRC, especially in young patients.

## Discussion

The annual incidence of sporadic CRC in young adults has been steadily increasing worldwide, but the tumor characteristics and mechanisms of tumor progression in yCRC have been less studied than those in oCRC (14). The microbiome and the ecosystem it creates have begun to attract extensive attention, owing to their important role in tumor development and excellent performance in early diagnosis and prognostic evaluation (15).

Homeostasis of the microbiome (including bacteria, viruses, and fungi) regulates body health, and dysbiosis can lead to disease (16). Accumulating evidence has shown that alterations in the abundance of the gut microbiome promote chronic inflammation and the production of carcinogenic metabolites that contribute to CRC formation (17). In addition, evidence shows that changes in the gut microbiome often occur in the early stages of colorectal carcinogenesis; therefore, microbiome changes may be used as biomarkers for early detection of CRC to improve screening strategies (7). By comparing stool samples collected from patients with CRC and healthy controls, many studies have reported increased levels of some gut microbiomes in patients with CRC (18), including *Fusobacterium nucleatum*, Enterotoxigenic *Bacteroides fragilis*, and *Escherichia coli*. In addition, some oral microbiomes, such as *Porphyromonas*, *Peptostreptococcus*, and *Parvimonas micra*, were also enriched in the stool of patients with CRC. However, the gut microbiome of elderly individuals differs from that of young individuals. As major risk factors (unhealthy diet, obesity, and sedentary lifestyle) increase in prevalence in young adults, their gut microbiome might change (19). Therefore, our study focused on exploring the microbiome composition and function of yCRC and establishing a microbiome signature to differentiate yCRC from oCRC.

The significant role of the gut microbiome in various human diseases, especially tumors, has been confirmed by many studies, but accumulating evidence indicates that tumor tissue also has a local type-specific microbiome involved in tumor progression and immune response (20). The diversity and composition of the tumor microbiome have been reported to influence pancreatic cancer outcomes through modulated immune responses (21). Cai et al. reported that a conserved intracellular bacterial profile was detected in breast cancer, which played an important role in promoting cancer metastasis (22). Studies of hepatocellular carcinoma (HCC) have found that patients with HCC exhibited imbalances in the tumor microbiome, with a higher abundance of *S. maltophilia*, which induced the expression of senescence-associated secretory phenotype in hepatic stellate cells and liver cirrhosis, contributing to the progression of HCC (23). Specific gut bacteria can invade CRC tumor tissue (24); therefore, the gut microbiome does not fully reflect the microbial composition in tumors, owing to differences in the invasive and colonizing abilities of different bacteria. Few studies have reported on the compositional and functional characteristics of the tumor microbiome in patients with yCRC.

In this study, we characterized the tumor microbiota composition of 39 yCRC or oCRC samples (discovery cohort) and validated it using an additional 78 samples (validation set) from human tissues, including CRC. 16S rRNA sequencing analysis was used to identify specific microbiomes in yCRC tissue, and IHC staining was used to analyze their correlation with immune cell infiltration and patient prognosis. Overall, the tumor microbial diversity of yCRC was lower than that of oCRC, indicating microbiome dysbiosis in patients with yCRC. Among all CRC samples, Proteobacteria and Firmicutes were the most abundant phyla; *Actinomyces*, *Diaphorobacter*, and *Schaalia cardiffensis* were the key microbiota in the yCRC group. In addition, *Actinomyces* taxa provided an excellent means to discriminate individuals with yCRC, and *Actinomyces* biomarkers could be used as a tool for distinguishing individuals with yCRC. Ma et al. performed 16S rRNA gene sequencing for 1038 samples and analyzed the fecal microbial composition and functional changes in yCRC, oCRC, and healthy controls. In contrast, our study characterized the tumor microbiota of yCRC and oCRC to explore their potential roles in the tumor immune microenvironment and tumorigenesis. The merit of our study is to report the microbial composition of the tumor microenvironment inhabiting yCRC and to clarify that yCRC and oCRC have distinct tumor microbial bases.

Ecosystems created by the resident microbiome have profound implications for human health and cancer development. Many microbiomes have been reported to be directly related to tumors. For example, *Helicobacter pylori* has been designated by the International Agency for Cancer Research as a carcinogen for gastric cancer, as it increases susceptibility to gastric cancer by activating the WNT/β-catenin pathway by transporting CagA to gastric epithelial cells (25). In addition to directly causing tumors, studies have suggested that microenvironmental dysregulation caused by interactions between microbiomes can promote tumor development by, for instance, secreting microbial virulence factors, participating in signal transmission, and influencing immune cell recruitment (26). Our research advances the understanding of the molecular mechanisms underlying *Actinomyces*- and gram-negative bacteria-associated CRC tumorigenesis. *Actinomyces* triggers the expression of several microbial- and immune-related genes, including TLR2, TLR4, and NF-κB, which promote CRC development by regulating inflammation and anti-tumor immunity. We further showed that the gram-positive bacteria *Actinomyces* was recognized by the specific receptor TLR2 and activated the downstream NF-κB pathway to regulate inflammation. Because *Actinomyces* co-occurs with various tumor microbiota and causes microbial dysbiosis in CRC, we speculated that *Actinomyces* activates the downstream TLR4/NF-κB pathway by regulating the occurrence of gram-negative bacteria. In addition, *Actinomyces* suppresses immune responses by inhibiting the infiltration of CD8+ T lymphocytes.

In conclusion, our study revealed a common state of tumor microbial dysbiosis in yCRC populations. Our findings may help identify novel contributors to yCRC pathogenesis, as well as evidence that the tumor microbiome influences the TME and immune response. Although further clinical and laboratory validation and mechanistic studies are necessary, our study highlights the need for further research on the potential association between the tumor microbiome and yCRC risk. We propose that the tumor-specific microbiome holds potential as a biomarker or as a new target for anti-tumor therapy.

## Conclusion

**Figure 7:**
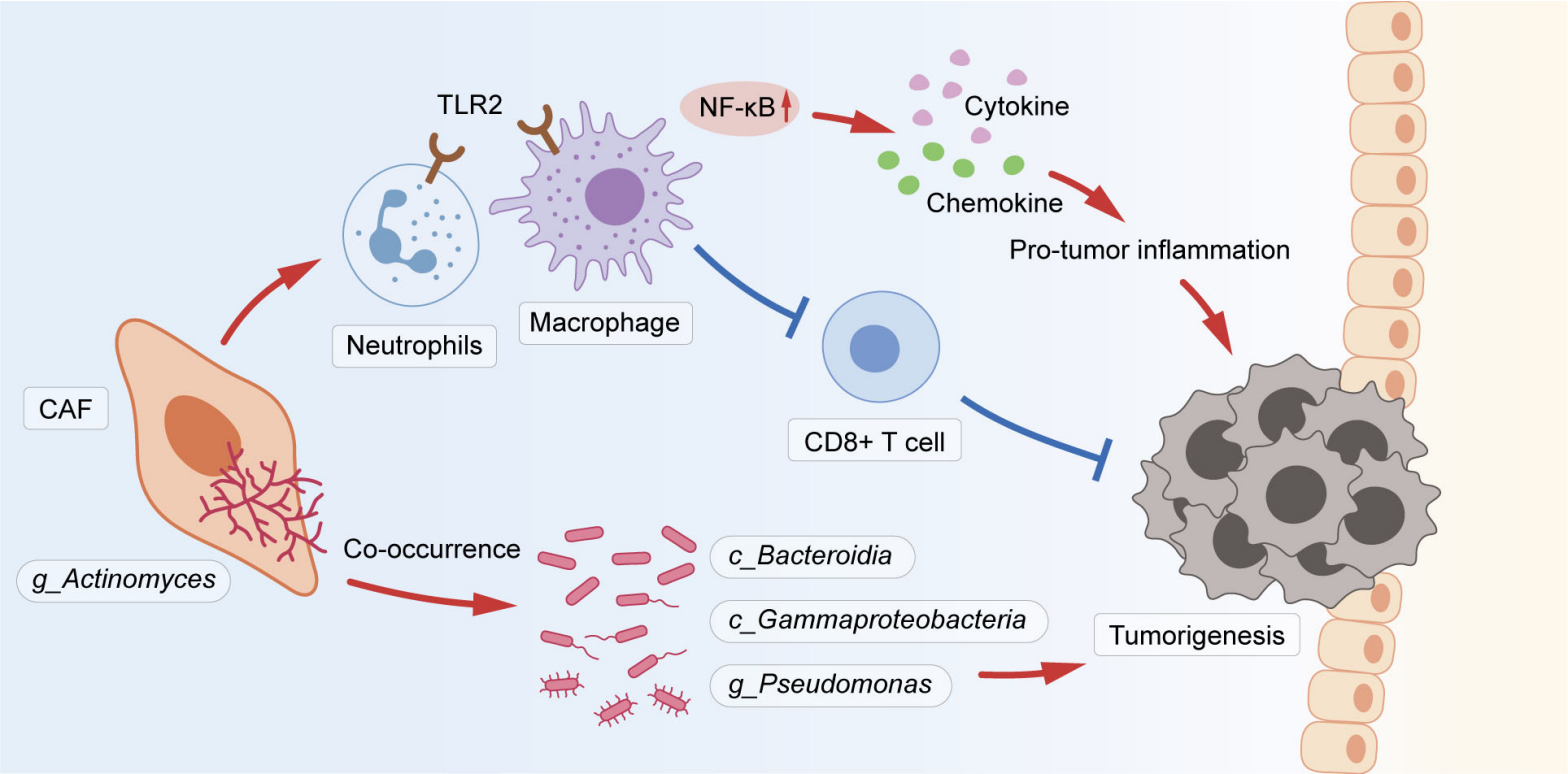
The schematic shows that *Actinomyces* in CRC resided in CAFs and co-occurred with various pro-tumor microbiota, including Bacteroidia, Gammaproteobacteria, and *Pseudomonas*. *Actinomyces* is recognized by TLR2 in neutrophils and macrophages and activates the downstream NF-κB pathway to regulate inflammation. Simultaneously, *Actinomyces* suppresses immune responses by inhibiting the infiltration of CD8+ T lymphocytes.

This study revealed that tumor microbial diversity was decreased in yCRC, and the genus *Actinomyces* was an important constituent of the yCRC microbiota. *Actinomyces* in CRC reside in CAFs and co-occur with various pro-tumor microbial taxa, including Bacteroidia, Gammaproteobacteria, and *Pseudomonas*. *Actinomyces* induces activation of the TLR2/NF-κB and TLR4/NF-κB pathways to promote inflammation and suppress anti-tumor responses by inhibiting the infiltration of CD8+ T lymphocytes (**Figure 7**).

## Data availability statement

The datasets presented in this study can be found in online repositories. The names of the repository/repositories and accession number(s) can be found below: BioProject under accession number PRJNA865279.

## Ethics statement

Ethical approval was obtained from Biomedical Ethics Committee of Ruijin Hospital, written informed consent was obtained from the patients/participants.

## Author contributions

All authors meet the authorship requirements. Conception and design: ZQX, WF, and AL. Development of methodology: ZL, FC, and SY. Acquisition of data (provided animals, acquired and managed patients, provided facilities, etc.): ZL, FC, and AT. Analysis and interpretation of data (e.g., statistical analysis, biostatistics, computational analysis): ZQX, ZL, and WF. Writing, review, and/or revision of the manuscript: ZQX, ZFX, and AL. Administrative, technical, or material support (i.e., reporting or organizing data, constructing databases): JH, WL, and YCZ. Study supervision: XS, JZ and, AL. Revision director: YPZ and XS. All authors contributed to the article and approved the submitted version.

## Funding

This study was supported by National Natural Science Foundation of China (81871933, 81802326). The funders of this project had no role in the design of the study and collection, analysis, and interpretation of data and in writing the manuscript.

## Conflict of interest

The authors declare that the research was conducted in the absence of any commercial or financial relationships that could be construed as a potential conflict of interest.

## Publisher’s note

All claims expressed in this article are solely those of the authors and do not necessarily represent those of their affiliated organizations, or those of the publisher, the editors and the reviewers. Any product that may be evaluated in this article, or claim that may be made by its manufacturer, is not guaranteed or endorsed by the publisher.

## Abbreviations

CRC, Colorectal cancer; yCRC, young-onset colorectal cancer; oCRC, old-onset colorectal cancer; TME, tumor microenvironment; IHC, Immunohistochemistry; FISH, Fluorescence *in situ* hybridization; ASVs, amplicon sequence variants; OTUs, operational taxonomic units; ACE, abundance-based coverage estimator; PCoA, principal coordinate analysis; Anosim, analysis of similarities; LDA, linear discriminant analysis; LEfSe, linear discriminant analysis effect size; PICRUSt, Reconstruction of Unobserved States; CAFs, cancer-associated fibroblasts; ROC, Receiver operating characteristic curves; AUC, Area under the curve; TLR, toll like receptors; HCC, epatocellular carcinoma.
