# Supplementary material for: Dysbiosis of human tumor microbiome and aberrant residence of Actinomyces in tumor-associated fibroblasts in young-onset colorectal cancer
Source: Front Immunol. 2022 Sep 2;13:1008975. doi: 10.3389/fimmu.2022.1008975 (PMC9481283; doi:10.3389/fimmu.2022.1008975)
Supplement: Supplementary file 1 [file DataSheet_1.docx]

**Table S1 Clinical characteristics for 16S rRNA sequencing analysis (discovery cohort).**

| Clinicopathologic parameters | Case | Age | | P- value |
| --- | --- | --- | --- | --- |
|  |  | yCRC (<50) | oCRC (≥50) |  |
| Total | 39 | 20 | 19 |  |
| Gender |  |  |  | 0.5273 |
| Male | 21 | 12 | 9 |  |
| Female | 18 | 8 | 10 |  |
| Histology |  |  |  | 0.0915 |
| Low | 6 | 1 | 5 |  |
| Moderate + High | 33 | 19 | 14 |  |
| BMI category (Mean ± SD) |  | 23.09 ± 3.087 | 24.76 ± 2.772 | 0.0832 |
| Neoadjuvant chemotherapy |  |  |  | 0.6948 |
| Received | 8 | 5 | 3 |  |
| Not received | 31 | 15 | 16 |  |
| Tumor location |  |  |  | 0.0958 |
| Right | 13 | 4 | 9 |  |
| Left | 26 | 16 | 10 |  |
| MSI status |  |  |  | 0.1060 |
| MSS | 35 | 16 | 19 |  |
| MSI-H | 4 | 4 | 0 |  |
| TMN stage |  |  |  | 0.1110 |
| I + II | 18 | 12 | 6 |  |
| III + IV | 21 | 8 | 13 |  |

**Table S2 Clinical characteristics for IHC analysis (validation set).**

| Clinicopathologic parameters | Case | Actinomyces abundance | | P- value |
| --- | --- | --- | --- | --- |
|  |  | High | Low |  |
| Total | 78 | 31 | 47 |  |
| Age |  |  |  | 0.0033 |
| <50 | 17 | 12 | 5 |  |
| ≥50 | 61 | 19 | 42 |  |
| Gender |  |  |  | 0.7439 |
| Male | 41 | 17 | 24 |  |
| Female | 37 | 14 | 23 |  |
| Histology |  |  |  | 0.1583 |
| Low | 22 | 6 | 16 |  |
| Moderate + High | 56 | 25 | 31 |  |
| Tumor location |  |  |  | 0.9746 |
| Right | 25 | 10 | 15 |  |
| Left | 53 | 21 | 32 |  |
| Tumor size |  |  |  | 0.2876 |
| ≥5 cm | 37 | 17 | 20 |  |
| <5 cm | 41 | 14 | 27 |  |
| TMN stage |  |  |  | 0.0082 |
| I + II | 41 | 22 | 19 |  |
| III + IV | 37 | 9 | 28 |  |

**Table S3 Probe sequence for hybridization**

| **Probe** | **Sequence** |
| --- | --- |
| Actinomyces | 5’- CGGTTATCCAGAAGAAGGGG -3’ |

**Table S4 Antibodies for IHC analysis**

| **Antibody (Source)** | **Catalog** | **Dilution for IHC** | **Manufacturer** |
| --- | --- | --- | --- |
| TLR2 (Rabbit) | ab213676 | 1:200 | Abcam, UK |
| TLR4 (Rabbit) | ab22048 | 1:200 | Abcam, UK |
| α-SMA (Rabbit) | ab32575 | 1:200 | Abcam, UK |
| CD8 alpha (Rabbit) | ab245118 | 1:200 | Abcam, UK |
| CD45 (Rabbit) | ab40763 | 1:200 | Abcam, UK |
| NF-kB p65 (Rabbit) | ab32536 | 1:200 | Abcam, UK |

**Table S5 Alpha diversity index by group.**

|  | yCRC_Median | yCRC_SD | CRC_Median | CRC_SD | yCRC_CRC_p.value | yCRC_CRC_q.value |
| --- | --- | --- | --- | --- | --- | --- |
| ace | 586.71 | 680.17 | 1251.48 | 676.82 | 0.003 | 0.007 |
| chao1 | 578.95 | 667.44 | 1256.73 | 675.35 | 0.002 | 0.007 |
| shannon | 3.93 | 1.30 | 4.66 | 1.52 | 0.063 | 0.106 |
| simpson | 0.82 | 0.12 | 0.83 | 0.11 | 0.673 | 0.673 |
| pielou_e | 0.46 | 0.11 | 0.46 | 0.11 | 0.354 | 0.442 |

**Table S6 Additional clinical information of discovery cohort.**

| Clinicopathologic parameters | Case | Actinomyces abundance | | P- value |
| --- | --- | --- | --- | --- |
|  |  | High | Low |  |
| Total | 39 | 16 | 23 |  |
| Age |  |  |  | 0.0225 |
| <50 | 20 | 12 | 8 |  |
| ≥50 | 19 | 4 | 15 |  |
| Gender |  |  |  | 0.0492 |
| Male | 21 | 12 | 9 |  |
| Female | 18 | 4 | 14 |  |
| Histology |  |  |  | >0.9999 |
| Low | 6 | 2 | 4 |  |
| Moderate + High | 33 | 14 | 19 |  |
| BMI category (Mean ± SD) |  | 23.78 ± 2.733 | 24.00 ± 3.262 | 0.8273 |
| Neoadjuvant chemotherapy |  |  |  | 0.2349 |
| Received | 8 | 5 | 3 |  |
| Not received | 31 | 11 | 20 |  |
| Tumor location |  |  |  | 0.1693 |
| Right | 13 | 3 | 10 |  |
| Left | 26 | 13 | 13 |  |
| MSI status |  |  |  | 0.6309 |
| MSS | 35 | 15 | 20 |  |
| MSI-H | 4 | 1 | 3 |  |
| T class (Invasion depth) |  |  |  | 0.6776 |
| T1-2 | 7 | 2 | 5 |  |
| T3-4 | 32 | 14 | 18 |  |
| N class (Lymph metastasis) |  |  |  | 0.7475 |
| N0 | 20 | 9 | 11 |  |
| N1-2 | 19 | 7 | 12 |  |
| M class (Liver metastasis) |  |  |  | 0.2864 |
| M0 | 25 | 13 | 22 |  |
| M1 | 4 | 3 | 1 |  |
| TMN stage |  |  |  | >0.9999 |
| I + II | 18 | 7 | 11 |  |
| III + IV | 21 | 9 | 12 |  |
