# Supplementary figures and images for: Dysbiosis of human tumor microbiome and aberrant residence of Actinomyces in tumor-associated fibroblasts in young-onset colorectal cancer
Source: Front Immunol. 2022 Sep 2;13:1008975. doi: 10.3389/fimmu.2022.1008975 (PMC9481283; doi:10.3389/fimmu.2022.1008975)

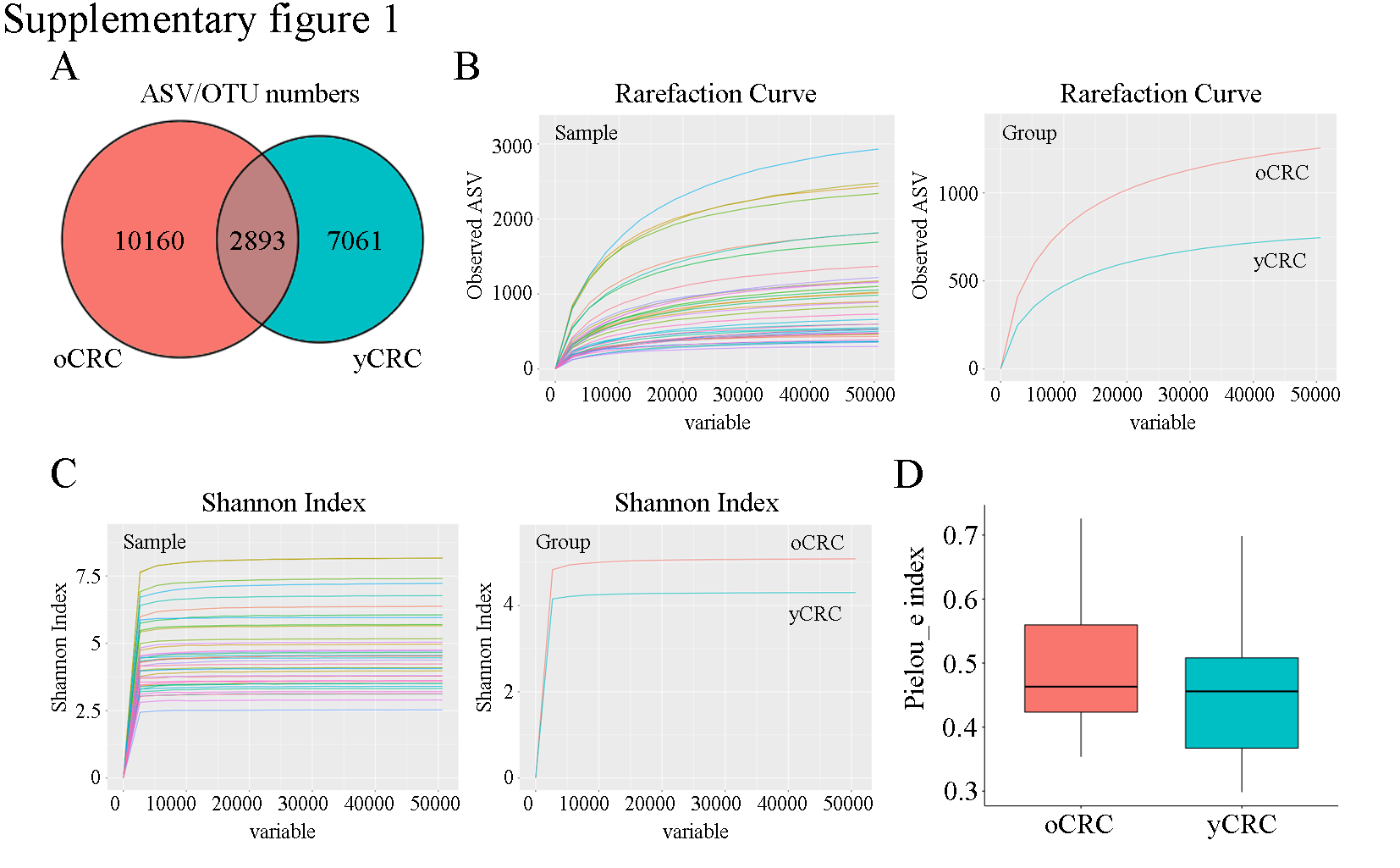

Supplement: Supplementary file 2 [file Image_1.tif]

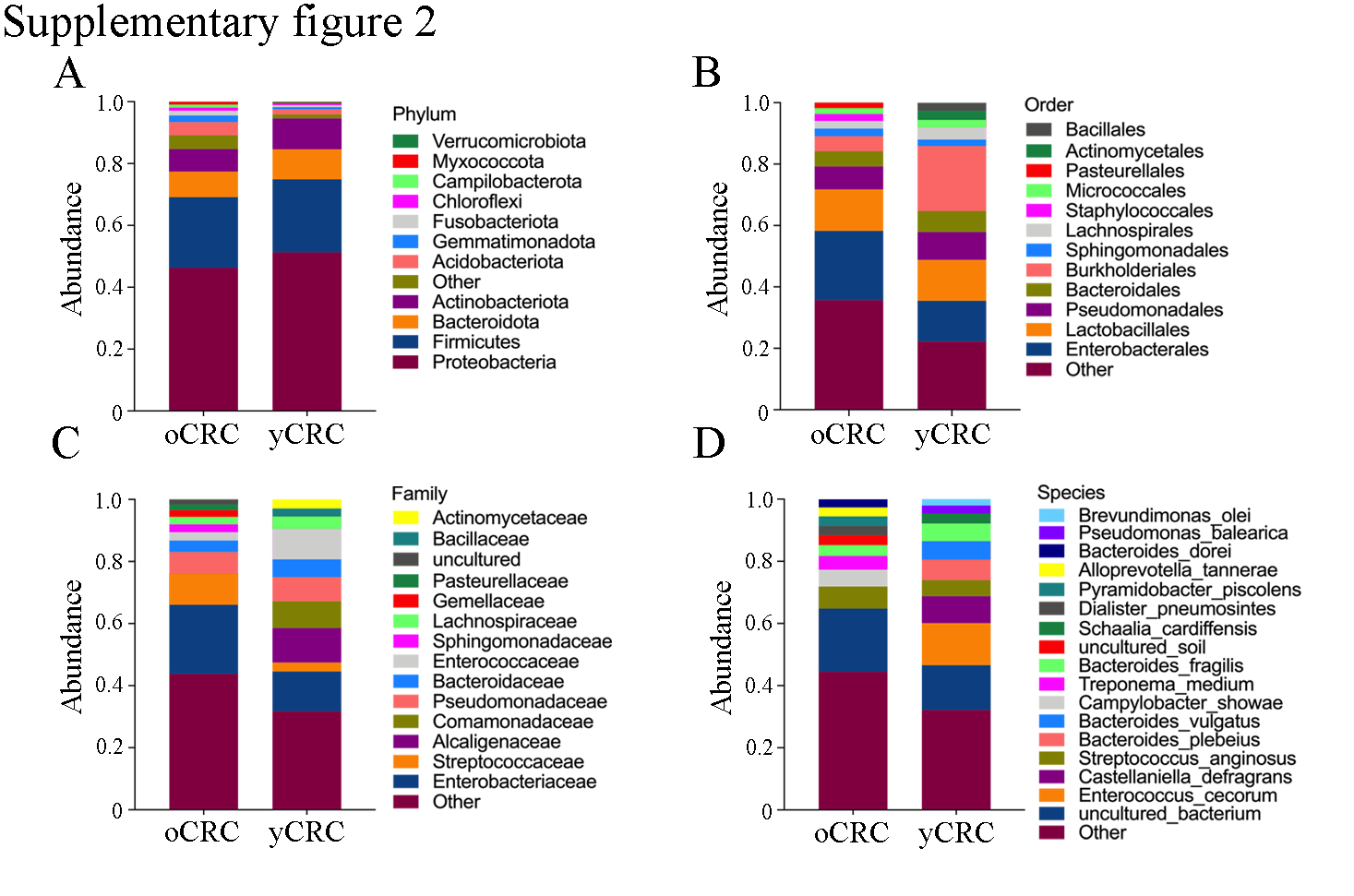

Supplement: Supplementary file 3 [file Image_2.tif]

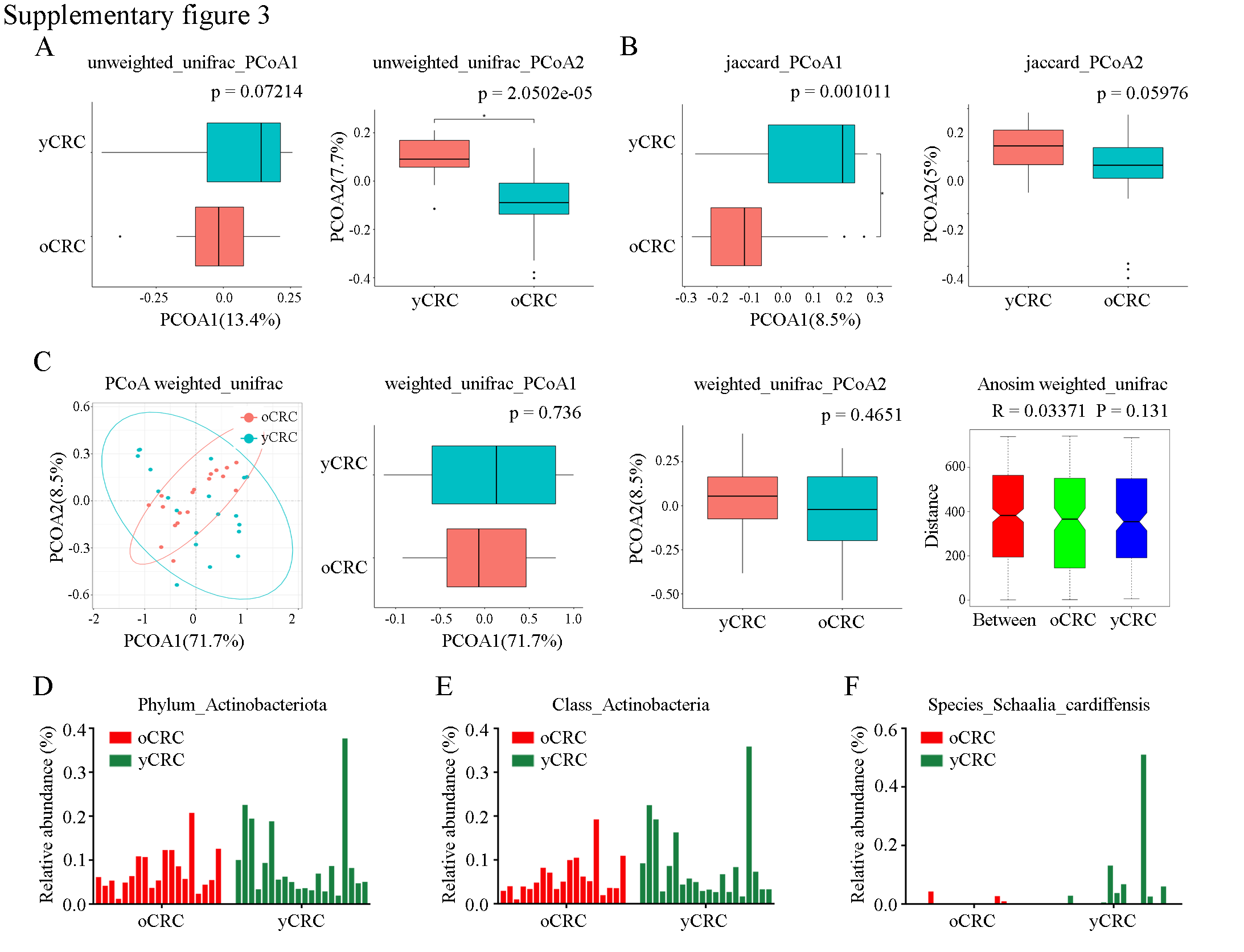

Supplement: Supplementary file 4 [file Image_3.tif]

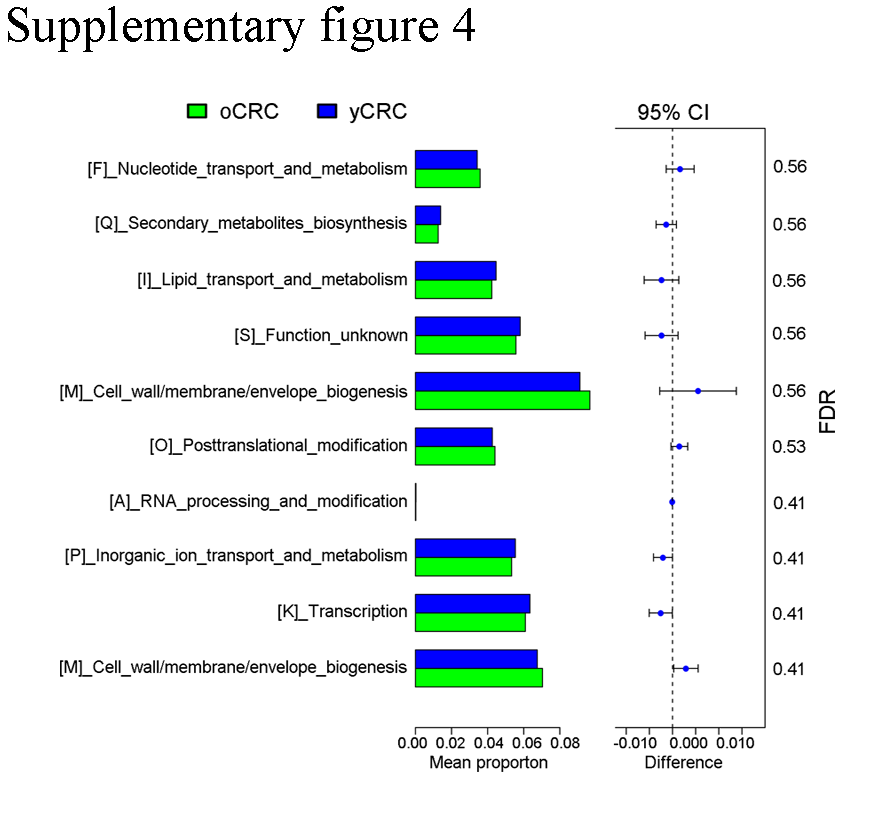

Supplement: Supplementary file 5 [file Image_4.tif]

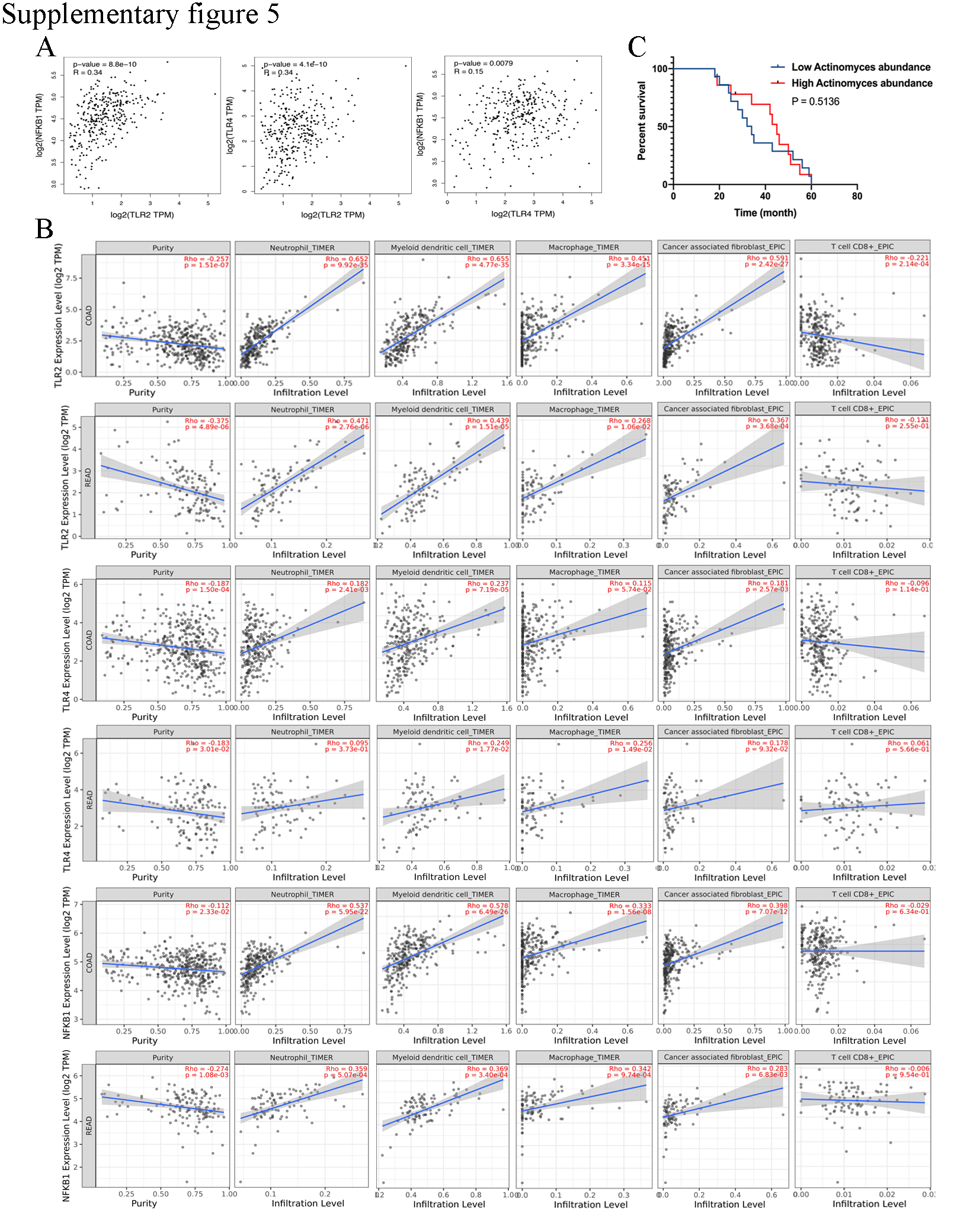

Supplement: Supplementary file 6 [file Image_5.tif]
