# Supplementary material for: Dysbiosis of human tumor microbiome and aberrant residence of Actinomyces in tumor-associated fibroblasts in young-onset colorectal cancer
Source: Front Immunol. 2022 Sep 2;13:1008975. doi: 10.3389/fimmu.2022.1008975 (PMC9481283; doi:10.3389/fimmu.2022.1008975)
Supplement: Supplementary file 7 [file Presentation_1.pdf]

Supplementary Methods

Extraction of genome DNA

Total genomic DNA from samples was extracted using CTAB or SDS method. DNA concentration was determined by Nanodrop. The purity and integrity could be evaluated through the 1% agarose gel electrophoresis.

Amplicon Generation

According to the concentration, DNA was diluted to 1ng/μl using sterile water. 16S /18S rRNA genes were amplified used the specific primer with the barcode. All PCR reactions were carried out in 30μL reactions with 15μL of High-Fidelity PCR Master Mix (New England Biolabs); 0.2μM of forward and reverse primers, and about 10 ng template DNA. The thermocycling conditions were as follows: 98 ℃ pre-degeneration for 1 minutes, denaturation at 98℃ for 10 seconds, annealing at 50 ℃ for 30 seconds, and extension at 72 ℃ for 30 seconds, with a total of 30 cycles, followed by a final elongation step at 72 ℃ for 5 minutes.

PCR Products quantification and qualification

Same volume of 1× loading buffer were mixed (contained SYBR green) with PCR products and electrophoresis were operated on 2% agarose gel for detection. Samples with bright main strip between 400-450bp were chosen for further experiments.

PCR Products Mixing and Purification

Amplicons were pooled in equal proportions and purified using TIANgel Purification Kit (TIANGEN Biotech). The purified product was used to prepare the Illumina DNA library.

Library preparation and sequencing

Sequencing libraries were generated using TIANSeq Fast DNA Library Prep Kit (illumina) (TIANGEN Biotech). The library quality was assessed on the Qubit@ 2.0 Fluorometer (Thermo Scientific) and Agilent Bioanalyzer 2100 system. At last, the library was sequenced on the Illumina platform using the 2 × 250 bp paired-end protocol.

DADA2 denoised

Microbiome bioinformatics were performed with QIIME 2 with slight modification according to the official tutorials. Briefly, raw sequence data were demultiplexed using the demux plugin following by primers cutting with cutadapt plugin(Martin et al., 2011). Sequences were then quality filtered, denoised, merged and chimera removed using the DADA2 plugin(Callahan et al., 2016).

Species Annotation

Species annotation was performed using QIIME2 software. For 16S/18S, the annotation database is Silva Database, while for ITS, it is Unite Database.

Phylogenetic Relationship Construction

In order to study phylogenetic relationship of each ASV and the differences of the dominant species among different samples(groups), multiple sequence alignment was performed using QIIME2 software.

Alpha Diversity

Alpha diversity is applied in analyzing complexity of species diversity for a sample through 6 indices, including Observed-species, Chao1, Shannon, Simpson, ACE, Good-coverage. All these indices in our samples were calculated with QIIME2 and displayed with R software.

Beta Diversity

Beta diversity analysis was used to evaluate differences of samples in species complexity, Beta diversity on both weighted and unweighted unifrac were calculated by QIIME2 software.

Cluster analysis was preceded by principal component analysis (PCA), which was applied to reduce the dimension of the original variables using the statpackage and ggbiplot package in R software (Version 3.6.2). Principal Coordinate Analysis (PCoA) was performed to obtain principal coordinates and visualize differences of samples in complex multi-dimensional data. A matrix of weighted or unweighted unifrac distances among samples obtained previously was transformed into a new set of orthogonal axes, where the maximum variation factor was demonstrated by the first principal coordinate, and the second maximum variation factor was demonstrated by the second principal coordinate, and so on. The three-dimensional PCoA results were displayed using QIIME2 package, while the two-dimensional PCoA results were displayed using ade package and ggplot2 package in R software (Version 3.6.2).

Functional gene prediction analysis

16S rRNA gene sequences were predicted in multiple functional databases using PICRUSt2, including KEGG (https://www.kegg.jp/), COG (https://www.ncbi.nlm.nih.gov/COG/) , MetaCyc (https://metacyc.org/), Pfam (http://pfam.xfam.org/) and TIGRFAM (http://tigrfams.jcvi.org/cgi-bin/index.cgi) etc.

Statistical analysis

To confirm differences in the abundances of individual taxonomy or function annotation between the two groups, Metastats and STAMP software was utilized. LEfSe analysis (LDA score threshold: 4) was used for the quantitative analysis of biomarkers within different groups. This method was designed to analyze data in which the number of species or function annotation is much higher than the number of samples and to provide biological class explanations to establish statistical significance, biological consistency, and effect-size estimation of predicted biomarkers. To identify differences of microbial communities between the two groups, ANOVA test were performed based on the Bray-Curtis dissimilarity distance matrices.

Reference

Callahan, B.J., McMurdie, P.J., Rosen, M.J., Han, A.W., Johnson, A.J., and Holmes, S.P. (2016). DADA2: High-resolution sample inference from Illumina amplicon data. Nat Methods 13, 581-583. doi: 10.1038/nmeth.3869.

Martin, A.J., Vidotto, M., Boscariol, F., Di Domenico, T., Walsh, I., and Tosatto, S.C. (2011). RING: networking interacting residues, evolutionary information and energetics in protein structures. Bioinformatics 27, 2003-2005. doi: 10.1093/bioinformatics/btr191.
